# Long-Term Neuroimaging Findings in a Murine Model of Human Extraparenchymal Neurocysticercosis

**DOI:** 10.1021/acsinfecdis.5c00431

**Published:** 2025-08-18

**Authors:** Alejandro Méndez, Agnes Fleury, Roger Carrillo-Mezo, Juan A. Hernández-Aceves, Montserrat Mejía-Hernández, Nelly Villalobos, Marisela Hernández, Raúl Bobes, Luis Concha, Juan J. Ortiz-Retana, Marta Romano, Pedro Tadao Hamamoto Filho, Gladis Fragoso, José Alejandro Espinosa-Cerón, Edda Sciutto

**Affiliations:** † Instituto de Investigaciones Biomédicas, 61738 Universidad Nacional Autónoma de México, 04510 México City, México; ‡ Instituto Nacional de Neurología y Neurocirugía, 14269 México City, México; § Facultad de Medicina Veterinaria y Zootecnia, Universidad Nacional Autónoma de México, 04510 México City, México; ∥ Instituto de Neurobiología, 73416 Universidad Nacional Autónoma de México, 76230 Querétaro, México; ⊥ Departamento de Fisiología, Biofísica y Neurociencias, Centro de Investigación y Estudios Avanzados del IPN, 07360 México City, México; # Department of Neurosciences and Mental Health, Botucatu Medical School, UNESP-Universidade Estadual Paulista, 18618-970 São Paulo, Brazil

**Keywords:** neurocysticercosis, cysticercosis, Taenia crassiceps, Taenia solium, diagnosis, HP10

## Abstract

Neurocysticercosis is caused by the establishment of *Taenia solium* cysticerci in the central nervous system. The extraparenchymal form (ExP-NCC) is the most severe clinical presentation that may remain asymptomatic for years. Current treatment involves cysticidal drugs (albendazole and/or praziquantel) combined with glucocorticoids to manage the associated neuroinflammation; however, only ∼30% of patients respond effectively. This highlights the need to improve therapeutic strategies. Herein, the experimental murine model of human ExP-NCC was further characterized to improve its usefulness in testing new therapies. In humans, cysts grow slowly in the basal cisterns of the subarachnoid space, and patients become symptomatic years after the infection. Thus, a long-term follow-up was performed by using magnetic resonance imaging (MRI) with sequences allowing volumetric analysis. MRI confirmed NCC in 77% of infected rats, all exhibiting extraparenchymal localization and persistently elevated levels of HP10, a marker of viable cysticerci. Imaging also enabled precise cyst localization and estimation of the parasite-occupied volume.

## Graphical abstract

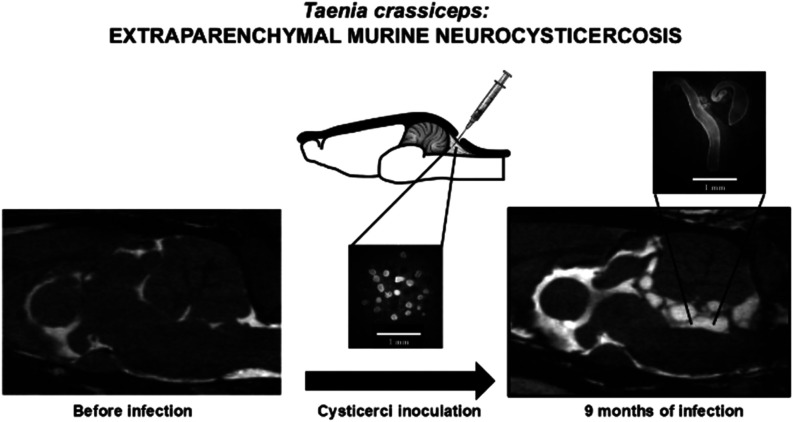

These findings strengthen the utility of this model for evaluating novel therapeutic approaches and provide valuable insights into the pathogenesis of severe forms of human NCC.

*Taenia solium* cysticercosis remains a significant public health concern in low- and middle-income countries across Latin America, Asia, and Africa, where inadequate environmental sanitation is common. Contributing factors include open defecation, insufficient drainage infrastructure, and free-roaming pigs that scavenge for food or are deliberately fed human feces.^1,2^ In humans, the metacestode is frequently established in the central nervous system (CNS), causing neurocysticercosis (NCC), the most severe form of the disease.^3,4^ In pigs, *T. solium* cysticerci can be found in skeletal muscles as well as in the CNS, causing only mild symptoms.^1^

Important advances have been made in human NCC epidemiology,^5^ diagnosis, vaccination,^6,7^ and treatment. However, the treatment of NCC caused by cysticerci located in the subarachnoid space or the ventricular space (ExP-NCC) remains a major unsolved challenge. Patients with ExP-NCC are treated with cysticidal drugs in combination with corticosteroids to control neuroinflammation and mitigate the risk of treatment-induced complications, such as intracranial hypertension and potentially fatal outcomes.^8–10^

Unfortunately, a single cycle of this combination for 10–15 days is efficient in only 30% of patients. The remaining 70% require multiple cycles of treatment or even endoscopic surgical interventions to remove the parasite when this is feasible.^11^ The complexity of the current treatment results in a progressive deterioration of the general health status of the patients without achieving the complete destruction or removal of the parasites.^12^ Currently, two cysticidal drugs—albendazole and praziquantel—are recommended for the treatment of human NCC; however, both drugs, whether used alone or in combination, show limited efficacy against the parasite in severe cases of ExP-NCC.^10^ New therapeutic candidates are currently being evaluated in animal models of NCC, showing promising results and offering renewed hope for improving treatment outcomes.^13–15^ In addition to limited parasiticidal efficacy, a major challenge in ExP-NCC management is controlling the associated neuroinflammation, particularly arachnoiditis and vasculitis, which can severely affect these patients.^16^ At present, glucocorticoids (GluCo), the most potent anti-inflammatory agents available, are coadministered with cysticidal drugs to control neuroinflammation.^17^ However, achieving therapeutic concentrations of glucocorticoids in the CNS requires high and prolonged dosing regimens.^18^ Such treatment is associated with significant adverse effects, including steroid-induced diabetes and Cushing’s syndrome, among others.^19,20^ Recent studies have also shown that glucocorticoids, beyond their established immunosuppressive effects, upregulate the expression of the immune checkpoint inhibitor PD-1,^21^ which may further compromise the efficacy of cysticidal therapy in ExP-NCC patients. These findings collectively underscore the urgent need to optimize treatment strategies for ExP-NCC.

The availability of cysticercosis experimental models that closely resemble this human form of the disease is a clear need for the search for new therapeutic options. Many models have been developed, such as murine intracerebral *Mesocestoides corti* infection,^22,23^ although the invasion ability of *M. corti* into the brain parenchyma limits its use as an ExP-NCC model.^24,25^ NCC caused in pigs by *T. solium* can be very useful for studying new treatment alternatives, leaving aside the difficulties inherent in the use of big animals.^26^ However, in naturally infected pigs, localization of the parasite in the basal subarachnoid space is extremely rare.^27^ The experimental rat model, established through intracranial infection with *T. solium* activated oncospheres, enables successful invasion of both parenchymal and extraparenchymal regions, thus providing a valuable platform for evaluating new therapeutic strategies. Nonetheless, the limited availability of *T. solium* oncospheres remains a significant barrier to use this model.^28^ In our research group, an accessible experimental model of ExP-NCC induced by the inoculation of *Taenia crassiceps* ORF cysticerci in the cisterna magna, developed by Hamamoto Filho et al., 2019 and 2021,^16,29^ is being explored. This reproducible model has extensive similarities with the human ExP-NCC, as previously demonstrated^30^ and seems useful for evaluating new treatments.

In this study, the usefulness of magnetic resonance imaging (MRI) sequences to diagnose and follow up murine ExP-NCC was demonstrated for the first time. The MRI sequences employed, let us identify the precise location, cysticerci stage, and volume occupied by the established parasites, which are valuable parameters to test the efficacy of new treatments.

## Results and Discussion

After inoculation of the parasites into the rat′s cisterna magna, long-term immunological and radiological monitoring was performed. HP10 levels were quantified from day 0 (beginning of the infection) until day 210 (7 months postinfection), with intermediate measurements at 2, 4, and 5 months (Figure 1).

**Figure 1:**
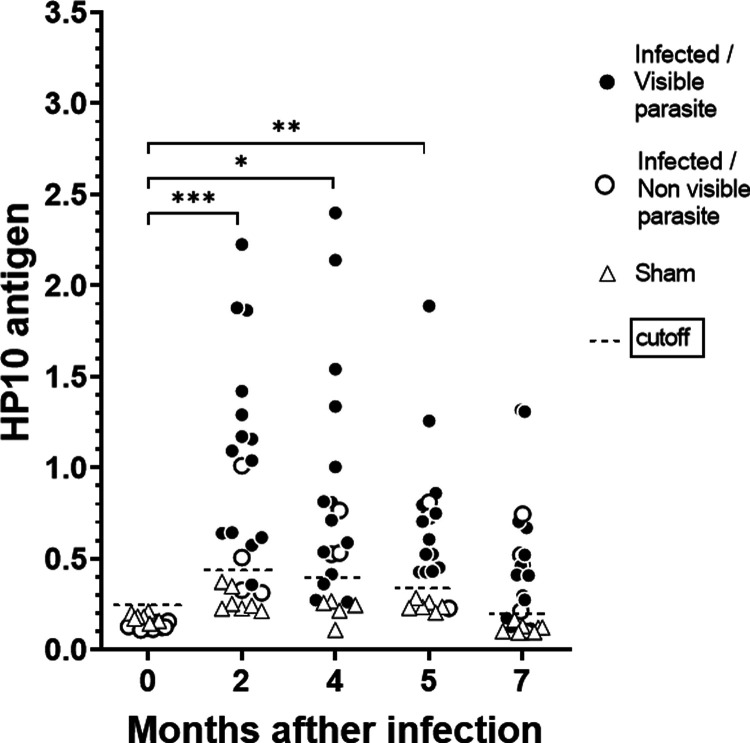
Levels of the HP10 antigen in experimentally infected rats with *T. crassiceps* cysts. HP10 antigen was detected in sera at 0, 2, 4, 5, and 7 months of infection. The cutoff value (dotted lines) was estimated based on the mean OD plus 3 SD from the sera of sham infected rats at each time of infection. The temporal evolution of HP10 levels within each group separately was evaluated using the Friedman test for related samples, considering only animals with complete data at all five time points (*n* = 8 for infected; *n* = 5 for sham). Asterisks indicate significant differences in the infected rat group over time (*** *p* ≤ 0.0001; ** *p* ≤ 0.01; * *p* ≤ 0.05). The black and white circles represent rats with the presence or the absence of cysticerci in MRI, respectively. Sham rats are represented with triangles.

Figure 1 shows the individual levels of the secreted HP10 antigen detected in each rat before and at various time points after cysticerci inoculation. HP10 levels increased significantly after infection in infected rats compared to the sham group at 2, 4, 5, and 7 months (Mann–Whitney test, *p* < 0.0001 at all 4 time points). Although HP10 levels began to decrease from 2 months on, they remained significantly elevated until 7 months. Increased HP10 levels are an indicator of the presence of vesicular cysticerci in the CNS, considering the results previously published.^31^

The presence of cysticerci in the CNS was detected at 9 months using magnetic resonance imaging (MRI) (Figure 2). MRI not only enabled the detection of parasites but also provided detailed information about their spatial distribution within the CNS. All scans were independently evaluated by two experienced neuroradiologists. From the 18 experimentally infected rats, cysticerci were distinguished in the extraparenchymal space of 14, resulting in a 77% success of the experimental infection. This value is slightly higher than the initially reported one by Filho et al., 2015,^32^ probably due to differences in the infectivity of the parasites employed.

**Figure 2:**
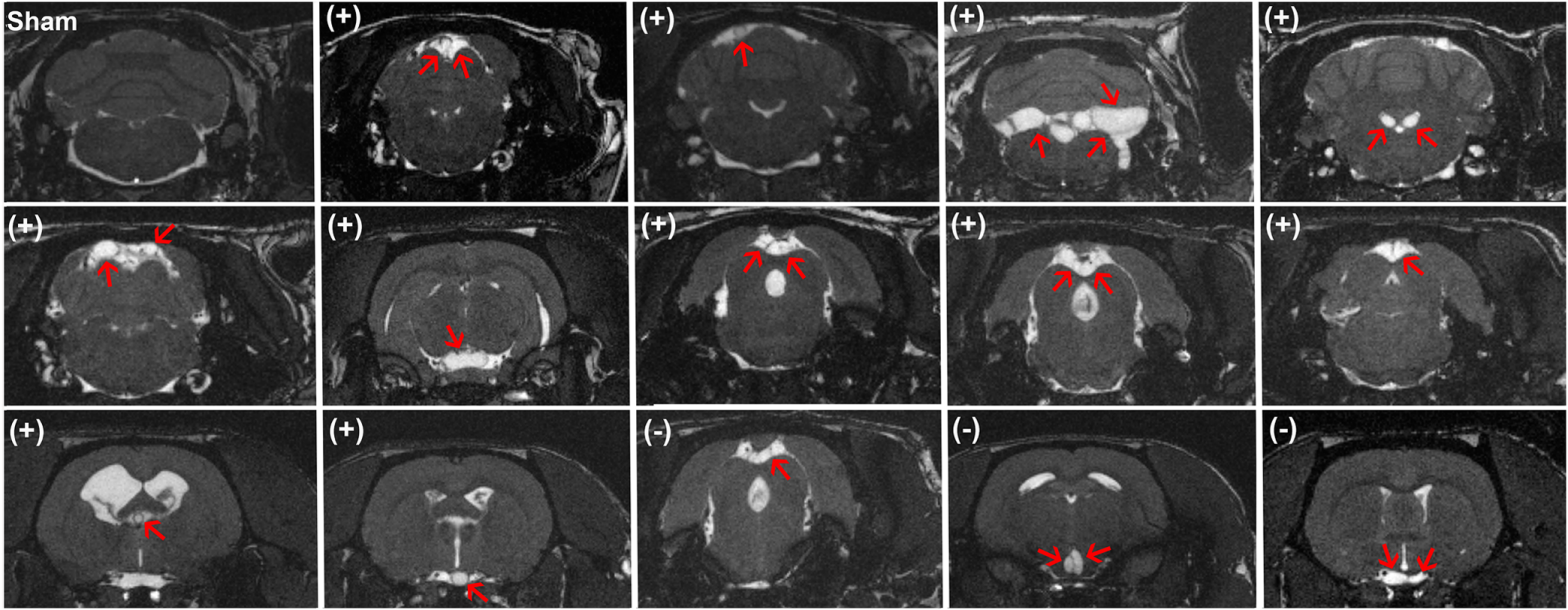
Coronal slice images of a representative Sham rat (upper left) and the 14 successfully infected rats in which cysticerci (marked with red arrows) were detected by MRI nine months after infection. The HP10 status (positive or negative) for each rat is indicated in parentheses of the corresponding image. Three rats with MRI confirmed that infection did not exhibit detectable levels of HP10.

Supporting Figure S1 shows the sagittal sections corresponding to each coronal slice from the 14 infected rats positive to MRI.

Figure 3 illustrates the extraparenchymal localization of cysticerci, as observed by MRI. Figure 3A presents coronal (upper panels) and sagittal (lower panels) MRI sections from four representative infected rats, showing the impact on the CNS, such as the hydrocephalus and/or mass effect. Figure 3B describes the anatomical locations of the cysticerci, while Figure 3C maps these sites within the ventricular system—including the lateral (LV), third (3 V), and fourth (4 V) ventricles—as well as the cisterna magna. Only a limited number of parasites were detected in the basal subarachnoid cisterns.

**Figure 3:**
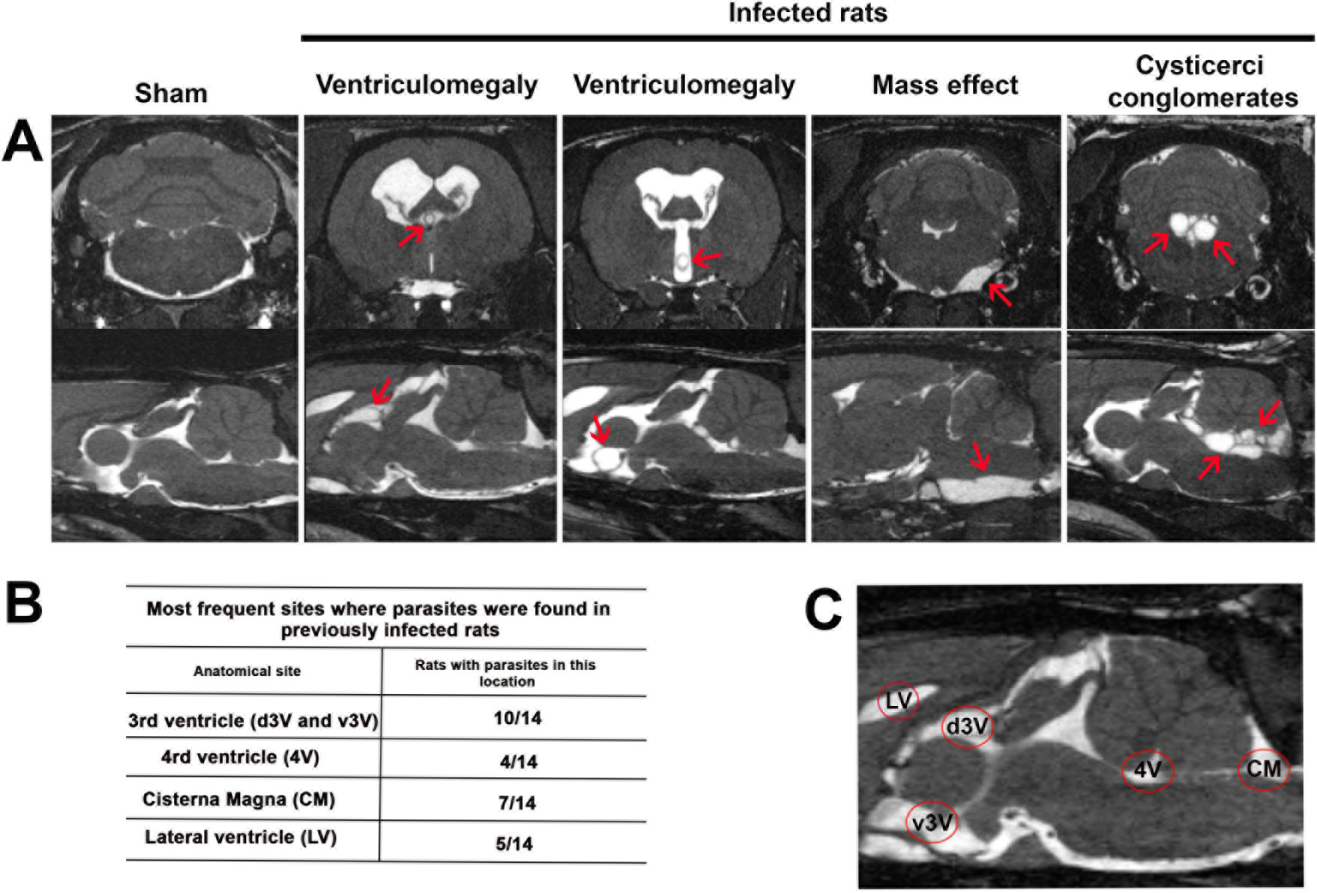
Hydrocephalus and/or mass effect observed in some ExP-NCC rats. (A) Representative images of the most frequent sites in the coronal (top) and sagittal slice (bottom) of the MRI, where parasites are detected in four representative images of infected rats. Single or conglomerate cysticerci causing ventriculomegaly and mass effect (marked with red arrows) can be distinguished. (B) Summary of the anatomical sites where cysticerci were detected by MRI and the frequency of their occurrence among the 14 infected rats. (C) Schematic representation of the most common parasite-hosting sites, as identified in sagittal slices: lateral ventricles (LV), third (3 V) and fourth (4 V) ventricles, and cisterna magna (CM).

The volume occupied by cysticerci in each rat was estimated to optimize conditions for monitoring the treatment response. Volumetric analysis was carried out on 14 rats in which the presence of parasites was confirmed by MRI. Figure 4 shows four coronal sections of a representative rat with 9 months of infection (A–D). Analysis of the entire area from the optic chiasma to the medulla of the 14 rats showed a mean parasitic volume of 17.29 ± 32.26 mm^3^ for females (*n* = 7) and 13.68 ± 20.94 mm^3^ for males (*n* = 7). The individual results of the volumetric analysis are listed in Table 1. No correlation was found between the volume occupied by cysticerci in the CNS and levels of HP10 (data not shown).

**Figure 4:**
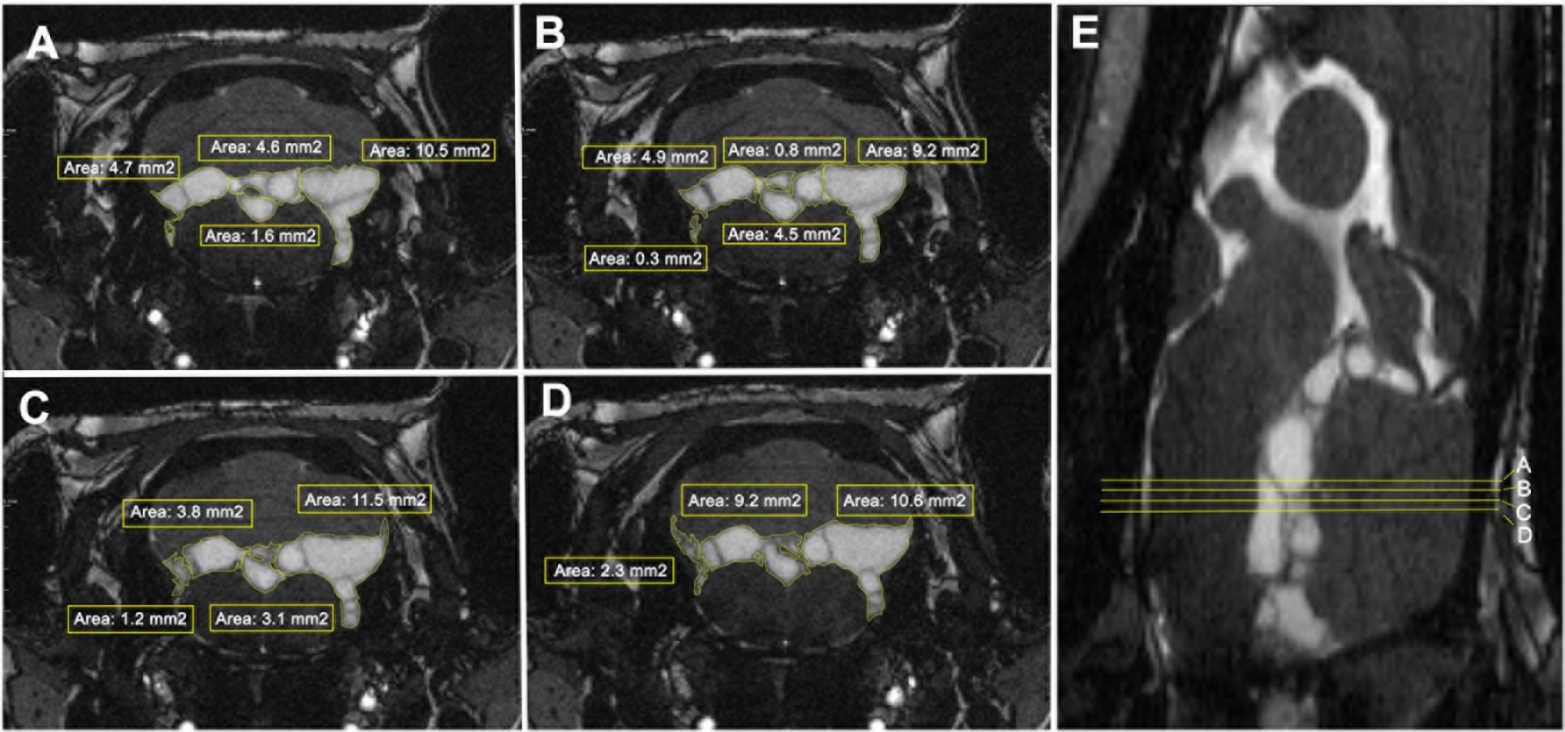
Analysis of parasite volume presented in 9 months – *T. crassiceps* infected rats (A–D). Four representative coronal section photographs of a rat with cysticerci are shown. The space occupied by the parasite (yellow lines) is marked with a tool of Weasis DICOM Viewer software in each image to determine the area in cm^2^ (the values contained for these images appear in the yellow boxes). (E) Sagittal section of the same analyzed rat that shows the location of the representative coronal sections (A–D). The entire analysis is performed on a total of 128 MRI images spanning from the beginning of the optic chiasma to the medulla.

**Table 1:** Signs of Infection in Male and Female Rats with 9 Months of Infection^a^

	HP10		MRI				
sex	serum	CSF	number	location	effects on CNS	CRP (pg/mL)	parasite vol (mm^3^)
Female	+	ND	conglomerate	cisterna magna; other basal SA cisterns	mass effect	460	5.19
Female	+	+	single/conglomerate	LV; other basal SA cisterns	ventriculomegaly with mass effect	490	7.17
Female	+	+	single/conglomerate	cisterna magna; other basal SA cisterns	mass effect	433	7.2
Female	+	ND	single/conglomerate	4 V; other basal SA cisterns	conglomerate of cysticerci without visible ventriculomegaly	530	1.26
Female	+	+	single/conglomerate	3 V; 4 V; LV; other basal SA cisterns	ventriculomegaly	421	6.31
Female	-	+	single/conglomerate	3 V; 4 V; other basal SA cisterns	conglomerate of cysticerci without visible ventriculomegaly	458	3.63
Female	+	ND	conglomerate	3 V; 4 V; LV; cisterna magna; other basal SA cisterns	ventriculomegaly with mass effect	475	90.3
Mean ± SD						465.33 ± 39.70	17.29 ± 32.26
Male	-	+	single/conglomerate	3 V; other basal SA cisterns	ventriculomegaly	451	8.4
Male	+	+	single/conglomerate	LV; 3 V; cisterna magna; other basal SA cisterns	ventriculomegaly with mass effect	485	60.425
Male	+	+	single	3 V; other basal SA cisterns	conglomerate of cysticerci without visible ventriculomegaly	293	3.4
Male	+	+	single/conglomerate	3 V; cisterna magna; other basal SA cisterns	ventriculomegaly with mass effect	495	11.35
Male	+	-	single/conglomerate	3 V; LV; cisterna magna; other basal SA cisterns	ventriculomegaly	501	8.27
Male	+	-	single	3 V; other basal SA cisterns	presence of cysticerci without visible effects	475	0.72
Male	-	ND	single	3 V; other basal SA cisterns	ventriculomegaly	435	3.17
Mean ± SD						431 ± 93.90	13.6764 ± 20.9434

a CRP: C-reactive protein; ND: not detected; LV: lateral ventricle; 3 V: third ventricle; 4 V: fourth ventricle; SA: subarachnoid.

Table 1 shows the main characteristics of 14 out of 18 infected rats in which the presence of the cysticerci was established based on the MRI. As it shows, a higher parasite volume was observed in female rats than in male rats, albeit the difference was not statistically significant due to the high-volume heterogeneity between animals. Only one female and two male rats tested negative for HP10 in serum, while two additional male rats were negative for HP10 in CSF. The presence of areas containing conglomerate cysticerci was observed, consistent with parasite reproduction by budding—a characteristic feature of *T. crassiceps*. All rats exhibited normal C-reactive protein levels.

The detection of vesicular cysticerci for up to 9 months of infection is relevant since this period represents an important part of the rat’s life, a finding in accordance with what occurs in human infection. Indeed, in humans, the persistence of cysticerci for years lodged out of the parenchyma of the CNS without symptoms is supported by a large series of cases reported in United Kingdom troops stationed in India.^33^ This probably indicates that a long period precedes the onset of the inflammatory reaction, which is the main cause of symptoms and medical consultation.^16^ A similar panorama occurs in this experimental murine model of extraparenchymal NCC, in which the establishment, growth, and reproduction of extraparenchymal cysticerci (Figures 1 and 2) did not modify the central or peripheral inflammatory profile assessed by protein C-reactive levels (Table 1), nor relevant behavioral changes during months after infection.^29^ However, parasites are not only successfully established after infection, but they also grow, as observed by the significant increase in parasite volume 9 months after infection (Table 1), a result in agreement with previously reported data.^34^

The absence of an inflammatory response observed in the infected rats is in accordance with the presence of vesicular parasites, a finding supported by the increased levels of the secreted antigen HP10 detected in the sera. A high concordance of parasite infection observed by MRI with serum HP10 levels was achieved. Indeed, considering MRI-based diagnosis as the “gold standard,” the HP10 assay had a sensitivity of 78.5% (11 of the 14 MRI-positive NCC rats were also positive for HP10). No parasites were identified in the CNS, either within or outside the brain parenchyma, in four experimentally infected rats. These animals were classified as negative based on MRI findings. However, three of them showed positive HP10 values. It is possible that cysticerci had migrated to the spinal cord beyond the field of view of the MRI, or that some parasites became lodged at the inoculation site near the probe entry point. Nevertheless, these cases were considered false positives, estimating the specificity of the HP10 detection method at 25%.

Another point that merits comment is the cysticerci distribution in the NCC experimentally induced. When NCC is induced by activated oncospheres as reported by Verastegui et al.,^28^ they can migrate through host brain tissues and develop into cysticerci in parenchymal and extraparenchymal regions, disregarding the site of inoculation. In the present study, fully developed cysticerci were inoculated into the rats’ cisterna magna, and all of them were detected by MRI in the extraparenchymal space. Unexpectedly, some of them were detected in the ventricular system, a shift that cannot be caused by normal circulation of the cerebrospinal fluid itself, which normally follows the opposite direction. This parasite distribution could be the result of their mobility toward sites more permissive to their growth and reproduction (Supporting Figure S2). Further studies will be conducted to better understand this phenomenon.

In human ExP-NCC infection, a 3D MRI sequence has been identified that allows the detection of cysticerci in the basal cisterns of the subarachnoid space with high definition.^35^ Herein, cysticerci were also detected with a high definition, allowing us to calculate the parasite-occupied volume in each infected rat. As previously shown in this study, parasite volume was associated with the development of ventriculomegaly and the mass effect. Therefore, this parameter was used as an indicator of the disease susceptibility.

Although parasite volume was generally higher in female rats compared to that in males, the difference was not statistically significant. The possibility of sexual dimorphism in the murine ExP-NCC model warrants further investigation, particularly considering findings from the intraperitoneal cysticercosis model in mice. In that model, marked sex-based differences were reported in immune and inflammatory responses, hormone levels, behavioral changes, and parasite load.^36,37^ While such dimorphism has not been clearly established in human disease, some evidence suggests that female ExP-NCC patients may present with a higher number of degenerating parasites and elevated cerebrospinal fluid (CSF) cellularity.^38^ These discrepancies may be attributed to differences in the anatomical constraints between the CNS and the peritoneal cavity or to distinct immune responses elicited within these two compartments.

The murine NCC-ExP model will allow the evaluation of new treatments for these severe forms of NCC. However, there is a limitation that even nine months after infection, the mice show no signs of exacerbated inflammation. It is possible that more time may be required for the parasitic infection to progress before the balance in this host-parasite relationship is lost and neuroinflammation is triggered. However, given the short lifespan of rats, this may not be sufficient.

## Conclusions

Overall, this study presents an experimental model of Exp-NCC that supported parasite growth and reproduction in 77.8% of experimentally infected animals nine months postinfection. At this time point, all of the infected rats harbored vesicular cysticerci. MRI enabled precise localization and volumetric assessment of the parasites within the CNS. The advancements in radiological characterization of cysticerci provided by this model enhance its relevance and utility for evaluating novel therapeutic strategies against the potentially fatal human form of ExP-NCC.

## Materials and Methods

### Rats

Eighteen Wistar rats (9 female and 9 male rats, from *Rattus norvegicus albinus* string) of 8–9 weeks of age were used for experimental infections with *T. crassiceps* ORF cysticerci. A group of 8 Sham rats (4 female and 4 male rats) was also included as controls in the experiment. All rats were maintained at 22 ± 3 °C under a 12/12 h light–dark cycle and with free access to water and food. Animals were weighed before and after experimental infection, and every 30 days. Experimental procedures used in this study were approved by the Institutional Committee for the Use and Care of Laboratory Animals of the Instituto de Investigaciones Biomédicas, UNAM (Number ID 6313). Rats were managed to minimize animal suffering and stress.

#### *Taenia crassiceps* Cysts and Inoculation

The procedure originally described by Filho et al.,^29^ with minor modifications previously published,^30^ was employed. Briefly, cysts of 400–500 μm in diameter were aseptically removed from the peritoneal cavity of previously infected mice (two or three months of *T. crassiceps* infection). Thirty cysts per rat, placed in a 20 cm long 0.010” inside diameter × 0.030” outside diameter Tygon Masterflex microbore transfer tubing (Cole-Parmer, Vernon Hills, IL) containing 50 μL of sterile phosphate-buffered saline (PBS), were inoculated in the cisterna magna of anesthetized rats under a stereotactic frame. During this process, the rat head was maintained at an 80–90° angle to identify the depressible surface between the occipital protuberance and the atlas spine. No signs of distress or locomotor abnormalities were observed.

Sham-operated rats underwent the same procedure, but they received cyst-free PBS. Serum samples were obtained by retro-orbital bleeding, following the NIH Office of Animal Care and Use guidelines,^39^ at different postinfection times.

### HP10 Ag-ELISA Assay

To assess the parasite viability when located in the brain of the infected rats, the level of the secreted HP10 antigen was detected in sera and cerebrospinal fluid (CSF) of noninfected and infected rats at 2, 4, 5, and 7 months postinfection by an Ag-ELISA assay following the procedure previously described.^30^ Briefly, the plates (Nunc, Rochester, New York, NY) were coated with monoclonal anti-HP10 antibody (MoAb) diluted to 1 μg/100 μL per well in borate-buffered saline (BBS), pH 8.2, and incubated overnight at 4 °C. The plate wells were blocked with bovine serum albumin (Roche, Ciudad de México, México) in PBS (1.0% w/v and 0.05% v/v Tween 20) and left for 60 min at room temperature. Between steps, the plates were washed four times with 0.15 M NaCl (w/v)/0.02% (v/v) Tween 20 in a Thermo Scientific Wellwash (Waltham, MA). Undiluted samples (85 μL/well) were added and incubated for 60 min at 37 °C. Biotinylated anti-HP10 MoAb diluted 1:1000 and horseradish-peroxidase-conjugated streptavidin (Zymed, San Francisco, CA) diluted 1:4000 were added consecutively, and the plates were incubated for 45 min at 37 °C. Then, tetramethylbenzidine (Invitrogen Carlsbad, CA) was added as the substrate. The reaction proceeded for 30 min at room temperature in the dark, and it was stopped by adding 100 μL of 0.2 M H2SO4 (Baker, Estado de Mexico, Mexico). Optical density (OD) was read at 450 nm in an ELISA processor Opsys MR Dynex Technology (Chantilly, VA).

### Magnetic Resonance Imaging (MRI)

*In vivo* MRI was performed on days 180 and 270 postinfection to detect the presence of extraparenchymal cysticerci. Acquisition protocols were carried out at the National Laboratory for Magnetic Resonance, located at the Instituto de Neurobiología. Imaging using a 7 T Bruker Pharmascan (Bruker Pharmascan 70/16US) was conducted. The animals were anesthetized with isoflurane and maintained at room temperature; the 40 mm transmission-receiver coil was used for the scanning. Images were acquired using a 3D sequence T2_TRUE_FISP 3D with TR/TE 4.4/2.2 ms en lugar de ms msec, 2 average, flip angle 30°, and a matrix 256 × 256 × 128, FOV of 34 mm × 34 mm × 19.5 mm, slice thickness 0.152 mm. Total scan time was 5 min 17 s. Software Weasis DICOM Viewer version 4.1.0. (Nicolas Roduit, https://github.com/nroduit/Weasis) was used for image analysis.

### Volumetric Assay

To estimate the parasite volume, MRI images were employed using a volumetric assay that consisted of analyzing the 128 MRIs of cysticerci that covered the complete optic chiasm-onset to medulla area, the site where cysticerci were found in previous MRI studies.^30^ Weasis DICOM Viewer version 4.1.0 software was employed.^40^ When a cysticercus is found in the MRIs, a tool of the program vectorizes the outline of the cysticercus and automatically calculates the area of the vectorized object (cysticercus). Each area obtained in each MRI was multiplied by the space between the MRI slices (152 μm). All values were added to estimate the total volume of the cysticercus.

### C-Reactive Protein (CRP) Measurement

As CRP is a classic inflammation indicator, serum CRP levels were assessed by a commercial enzyme-linked immunosorbent assay kit (Catalog DY1744) from R&D systems (Company, Minneapolis, MN) in noninfected and infected rats at 2, 4, 5, and 7 months postinfection. The procedure was performed according to the manufacturer’s directions.

### Statistical Analysis

Data were first tested for a normal distribution using the Kolmogorov–Smirnov test and tested for homogeneity of variance using Bartlett’s method. To evaluate differences in serum HP10 antigen levels between infected and control (sham) rats over time, two statistical analysis were performed. First, comparisons between groups were made using the Mann–Whitney *U* test due to the nonparametric nature of the data. The temporal evolution of HP10 levels within each group separately was performed using the Friedman test, considering infected animals with complete data at all five times (*n* = 8) for related samples, followed by the Dunn test. All statistical analyses were performed with Prism version 9.0 (GraphPad Software Inc., San Diego, CA). Results were considered statistically significant at *p* ≤ 0.05.

## Supplementary Material



## Notes

Sagittal MRI sections of the 14 infected rats in which cysticerci were detected nine months after infection, corresponding to Figure 1 (Figure S1); flux direction of cerebrospinal fluid and the hypothetical flux direction of parasites during inoculation in the murine model of extraparenchymal neurocysticercosis (Figure S2) (PDF)

## Abbreviations

NCC: neurocysticercosis
Exp-NCC: extraparenchymal neurocysticercosis
FIESTA: fast imaging employing steady-state acquisition
MRI: magnetic resonance imaging
GluCo: glucocorticoids
CNS: central nervous system
PD-1: programmed cell death protein 1
ORF: open reading frame
OD: optical density
LV: lateral ventricles
3 V: third ventricle
4 V: fourth ventricle
CM: cisterna magna
DICOM: digital imaging and communications in medicine
CSF: cerebrospinal fluid
CRP: C-reactive protein
ND: not detected
Ag ELISA: enzyme-linked immunosorbent assay
MoAb: monoclonal antibody
BBS: borate-buffered saline
ANOVA: analysis of variance
