# Supplementary material for: Long-Term Neuroimaging Findings in a Murine Model of Human Extraparenchymal Neurocysticercosis
Source: ACS Infect Dis. 2025 Aug 18;11(9):2534–41. doi: 10.1021/acsinfecdis.5c00431 (PMC12442056; doi:10.1021/acsinfecdis.5c00431)
Supplement: Supplementary file 1 [file id5c00431_si_001.pdf]

Supporting Information

Manuscript title:
Long-term neuroimaging findings in a murine model of human extraparenchymal neurocysticercosis

Authors:
Alejandro Méndez[1], Agnes Fleury[1,2], Roger Carrillo-Mezo[2], Juan A. Hernández-Aceves[1], Montserrat Mejía-Hernández[1], Nelly Villalobos[3], Marisela Hernández[1], Raúl Bobes[1], Luis Concha[4], Juan J. Ortiz-Retana[4], Marta Romano[5], Pedro Tadao Hamamoto Filho[6], Gladis Fragoso[1], José Alejandro Espinosa-Cerón[1]*, Edda Sciutto[1]*

Affiliations:

[1]Instituto de Investigaciones Biomédicas, Universidad Nacional Autónoma de México, México City, México.
[2]Instituto Nacional de Neurología y Neurocirugía, México City, México.
[3]Facultad de Medicina Veterinaria y Zootecnia, Universidad Nacional Autónoma de México, México City, México.
[4]Instituto de Neurobiología, Universidad Nacional Autónoma de México, Querétaro 76230, México.
[5]Departamento de Fisiología, Biofísica y Neurociencias, Centro de Investigación y Estudios Avanzados del I.P.N, México City, México.
[6]Department of Neurosciences and Mental Health, Botucatu Medical School, UNESP-Universidade Estadual Paulista, São Paulo 18618-687, Brazil.

***Corresponding author's email address:**

José Alejandro Espinosa-Cerón: alexec2803@gmail.com
Edda Sciutto: edda@unam.mx

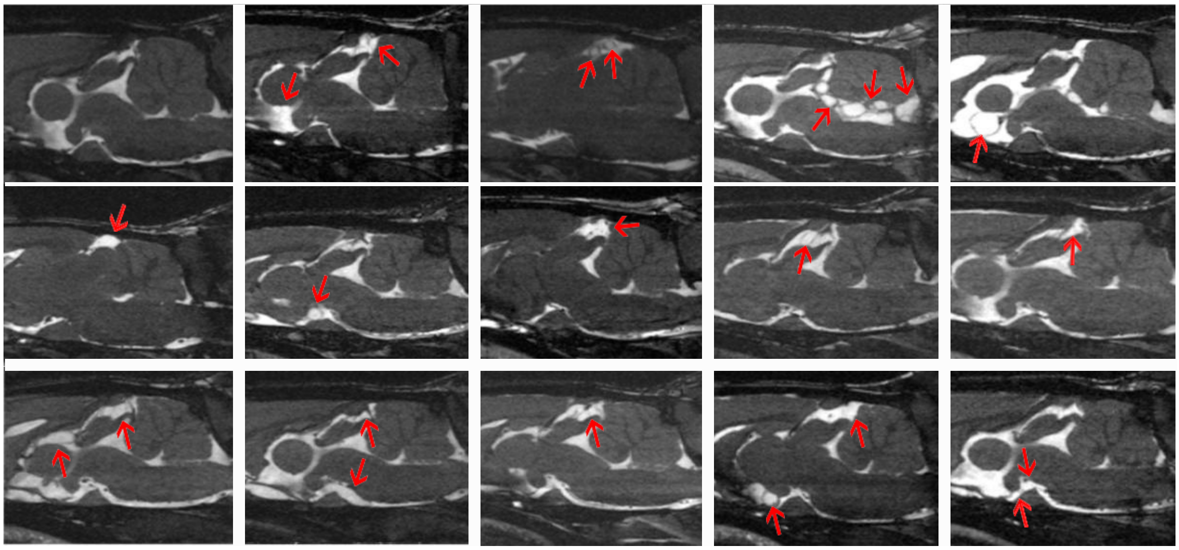

Supplementary figure S1. Sagittal MRI sections of the 15 rats that were included in the experiment to detect extraparenchymal neurocysticercosis nine months of infection. The MRI images correspond to each coronal slice which is shown in Figure 1B. Cysticerci are indicated with red arrows. In parentheses, it is indicated whether the rat had a positive or negative HP10 value. As a control, the first image corresponds to a representative Sham rat.

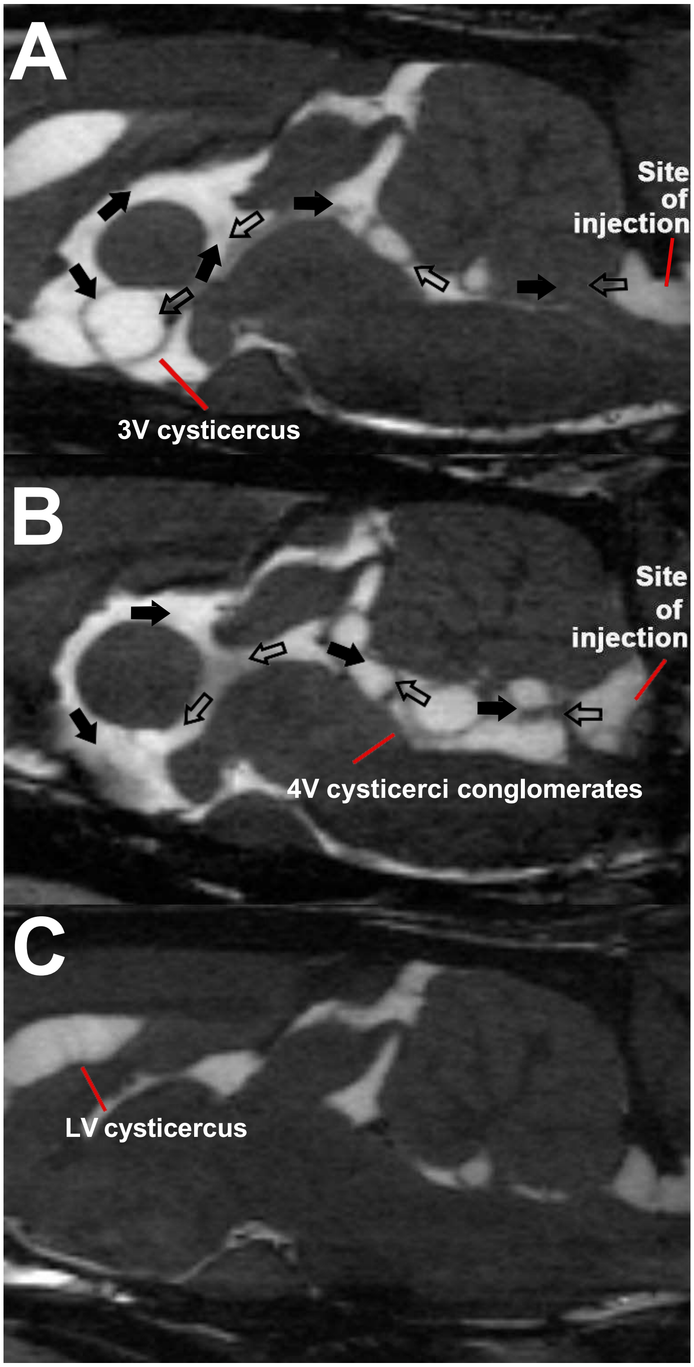

Supplementary figure S2. Flux direction of cerebrospinal fluid and the hypothetic flux direction of the parasites during inoculation in the murine model of extraparenchymal neurocysticercosis. Representative MRI sagittal sections from three rats with predominantly cysticerci in (A) the third ventricle, (B) the fourth ventricle, and (C) the lateral ventricles are shown. CSF circulation is illustrated with black arrows. The proposed direction that the cysticerci travel to reach their development site in the ventricles is indicated with unfilled arrows. Cysticerci are marked with red arrows. Red lines indicate the injection site of the parasites (Cisterna Magna).
